# Innovative use of a self-expanding valve for valve-in-valve transcatheter mitral valve replacement: experience from a four-year single-center study

**DOI:** 10.3389/fcvm.2023.1137663

**Published:** 2023-06-12

**Authors:** Yuehuan Li, Ruobing Lei, Jiawei Zhou, Kaisheng Wu, Jinglun Shen, Zhihui Zhu, Jiangang Wang, Haibo Zhang

**Affiliations:** ^1^ Department of Cardiac Surgery, Beijing Anzhen Hospital, Capital Medical University, Beijing, China; ^2^ Chevidence Lab of Child & Adolescent Health, Department of Pediatric Research Institute, Children’s Hospital of Chongqing Medical University, Chongqing, China; ^3^ Department of Medicine IV, LMU University Hospital, Ludwig Maximilian University of Munich, Munich, Germany

**Keywords:** valve-in-valve transcatheter mitral valve replacement, failed mitral bioprosthetic valves, transapical approach, health-related quality of life outcomes, Kansas city cardiomyopathy questionnaire-12

## Abstract

**Background:**

Valve-in-valve transcatheter mitral valve replacement (ViV-TMVR) is a minimally invasive option for patients with bioprosthetic mitral valve failure. Since January 2019, our center has been using a new innovative option, J-Valve, to treat patients with bioprosthetic mitral valve failure who were at high risk for open heart surgery. The aim of this study is to explore the effectiveness and safety of J-Valve and report the results from the four-year follow-up period of the innovative application of the transcatheter valve.

**Methods:**

Patients who underwent the ViV-TMVR procedure between January 2019 and September 2022 in our center were included in the study. J-Valve™ system (JC Medical Inc., Suzhou, China) with three U-shape grippers was used for ViV-TMVR via transapical approach. Data on survival, complications, transthoracic echocardiographic results, New York Heart Association functional class in heart failure, and patient-reported health-related quality of life according to the Kansas City Cardiomyopathy Questionnaire-12 (KCCQ-12) were collected during the four-year follow up.

**Results:**

Thirty-three patients (mean age 70.1 ± 1.1 years, 13 men) were included and received ViV-TMVR. The surgery success rate was 97%: only one patient was converted to open-heart surgery due to intraoperative valve embolization to the left ventricle. During the first 30 days all-cause mortality was 0%, risk of stroke 2.5% and risk of mild paravalvular leak 15.2%; mitral valve hemodynamics improved (179.7 ± 8.9 at 30 days vs. 269 ± 49 cm/s at baseline, *p* < 0.0001). Median time from operation to discharge was six days, and there were no readmissions within 30 days from operation. The median and maximum follow-up durations were 28 and 47 months, respectively; during the entire follow-up, all-cause mortality was 6.1%, and the risk of cerebral infarction 6.1%. Cox regression analysis did not identify any variables significantly associated with survival. The New York Heart Association functional class and the KCCQ-12 score improved significantly compared with their preoperative values.

**Conclusion:**

The use of J-Valve for ViV-TMVR is safe and effective with a high success rate, low mortality and very few associated complications, representing an alternative surgical strategy for the elderly, high-risk patients with bioprosthetic mitral valve failure.

## Introduction

The use of mechanical prostheses requires long-term anticoagulation, leading to an increased risk of bleeding in patients (1). In contrast, bioprosthetic valves are associated with a low rate of thrombosis and do not require lifetime anticoagulation. As a result, the preference for bioprosthetic valves has increased among patients with mitral valve disease over the past two decades (1). Guidelines for the treatment of valvular heart disease (2, 3) recommend patient preference as the primary criterion in selecting the type of prosthetic valve, further contributing towards the use of bioprosthetic valves. However, because of the limited durability, bioprosthetic valves need to be replaced over time. One study has shown that up to one-third of patients need to receive redo surgical treatment for mitral valve replacement (4). Since redo open-heart valve replacement surgery poses a risk of perioperative death (5), transcatheter valve-in-valve implantation technologies with less invasive alternatives for the treatment of bioprosthetic heart valve failure, which have been proven to be associated with lower risk of death, lower periprocedural morbidity, lower risk of complications, and lower need of resources, have gradually emerged since 2007 (4, 6).

However, valve-in-valve transcatheter mitral valve replacement (ViV-TMVR) still faces several challenges. Sapien 3 (Edwards Lifesciences Inc., Irvine, CA, USA) is the only transcatheter heart valve (THV) currently approved by the US Food and Drug Administration for ViV-TMVR and was also approved for the market by the Chinese National Medical Products Administration in 2020. Studies have also reported some disastrous complications associated with ViV-TMVR, such as valve migration or embolization and left ventricular outflow tract (LVOT) obstruction (7–11).

To avoid the above-mentioned complications, and also due to the fact that Sapien 3 was not available in China until 2019, we attempted to perform ViV-TMVR without changing the valve and transmitter structure using reverse-loaded J-Valve (JC Medical Inc., Suzhou, China), and concluded a standardized valve release process. J-Valve is a self-expanding transcatheter valve consisting of three U-shaped grippers, a crowned nitinol stent, porcine aortic valve leaflets, and an inner liner skirt. It was approved by China's Food and Drug Administration in 2017 with a dual indication for aortic stenosis and aortic regurgitation. Unlike cylindrical balloon-expanded valves, the anchoring of the J-Valve does not rely solely on radial forces. The three U-shaped grippers limit the movement of the valve towards the left atrium under left ventricular pressure, reducing the risk of valve migration or embolization. In addition, the three U-shaped grippers facilitate accurate commissural alignment, and the combined crowned stent and inner liner skirt both reduce the risk of left ventricular outflow tract obstruction and prevent the occurrence of paravalvular leaks.

In this study, we present the outcomes among elderly, high-risk patients with bioprosthetic mitral valve failure who were managed successfully by ViV-TMVR using J-Valve via transapical approach.

## Methods

### Patients

We included patients with failed surgical bioprosthetic valves who underwent ViV-TMVR in Beijing Anzhen Hospital (Capital Medical University, Beijing, China) between January 2019 and September 2022.

Preoperative electrocardiographic gated multislice computed tomographies (CT) were performed for all patients. Each patient was independently evaluated by at least two cardiac surgeons before the operation. We included patients aged ≥60 years for whom conventional redo valve surgery was associated with high risks (Society of Thoracic Surgery (STS) Risk Score or European System for Cardiac Operative Risk Evaluation (EuroSCORE) II of ≥8). Patients with the following conditions were excluded: (1) Combined moderate to severe mitral valve perivalvular leak (PVL); (2) History of stroke over the past three months; (3) Presence of left atrial or appendage thrombus; (4) Failed bioprosthetic valve type of ≤23 mm; (5) Presence of infective endocarditis; (6) Presence of LVOT obstruction; or (7) Combined multiple organ system failure or other diseases associated with a life expectancy of less than one year.

Written informed consent was obtained from all participants. The study was conducted in accordance with the Declaration of Helsinki (2013 revision). The study design was approved by the Ethics Review Committee of Beijing Anzhen Hospital (No. 2022083X).

### Procedure details

**Figure 1:**
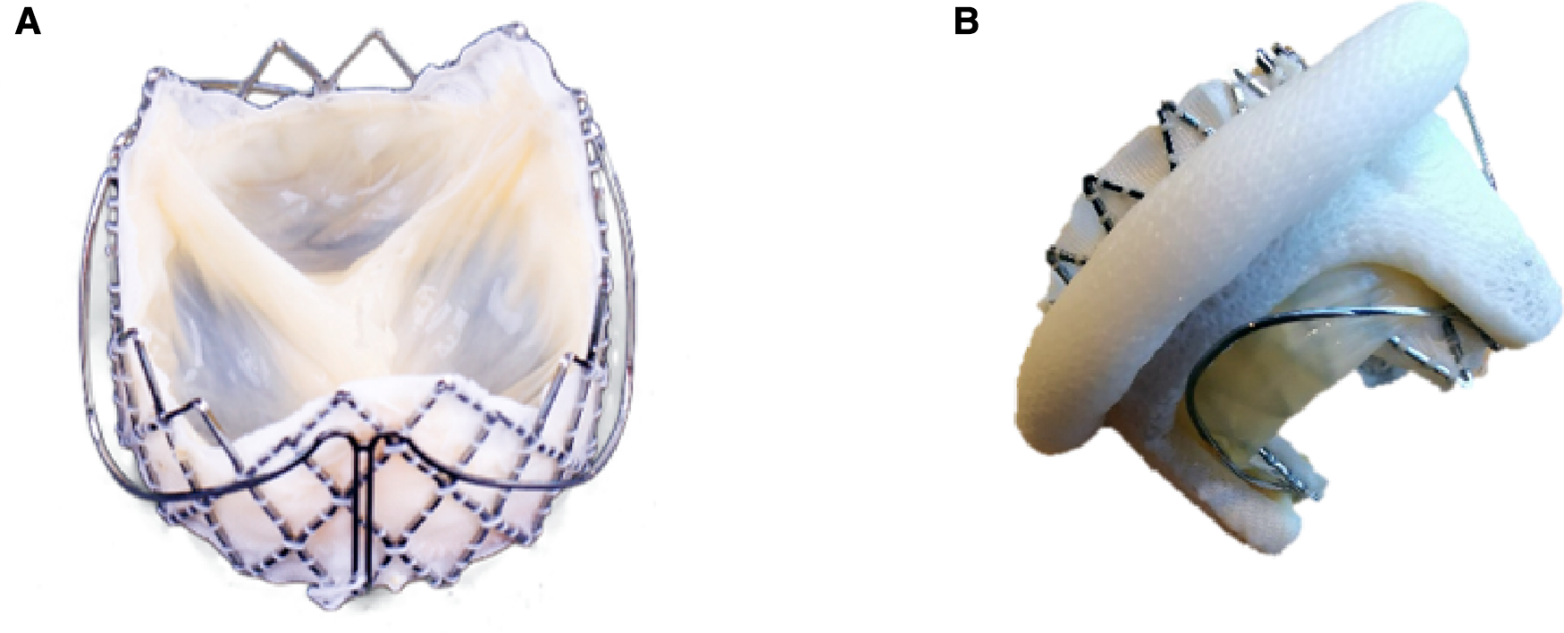
Features of J-Valve. The J-Valve is a self-expanding transcatheter valve with three U-shaped grippers and notches (**A**). The grippers help achieve commissure alignment (**B**) and further reduce the risk of left ventricular outflow tract obstruction.

The procedure was performed in a hybrid operating room. Transesophageal echocardiography (TEE) was performed under general anesthesia to determine the presence of left atrial appendage thrombus and mitral valve PVL. All patients were treated with a transapical approach using the J-Valve™ System (Figure 1). The J-Valve was reverse loaded and sized according to the measured internal diameter of the failed bioprosthetic valve. The THVs' oversize ratio ranged from 5%–10%.

**Figure 2:**
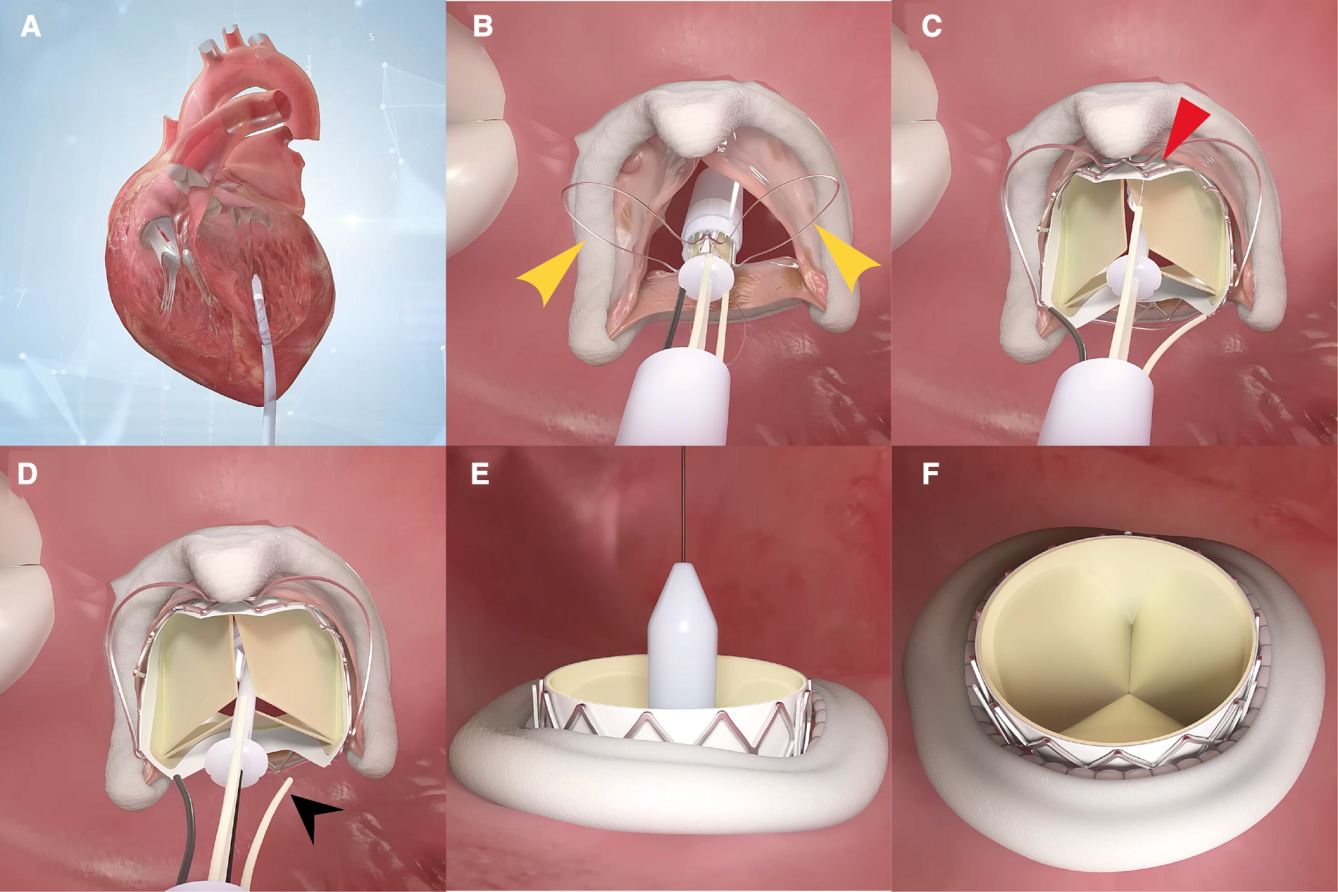
A step-by-step demonstration of the procedure of how J-valve functions. The conveyor enters the left ventricle along the extra-stiff guidewire (**A**), then the three U-shaped grippers are first released and subsequently staggered them between three struts of the bioprosthetic valve (**B**). The valve is then slowly released under rapid ventricular pacing (**C**). Subsequently, the conveyor anchor device is controlled to de-load the valve (**D**). The conveyer is withdrawn, and the guidewire retained (**E**). If balloon valvuloplasty is not required, the guidewire is withdrawn. The ideal implantation depth is 80% of the transcatheter heart valve stent frame in the left ventricle and 20% in the left atrium (**F**).

**Figure 3:**
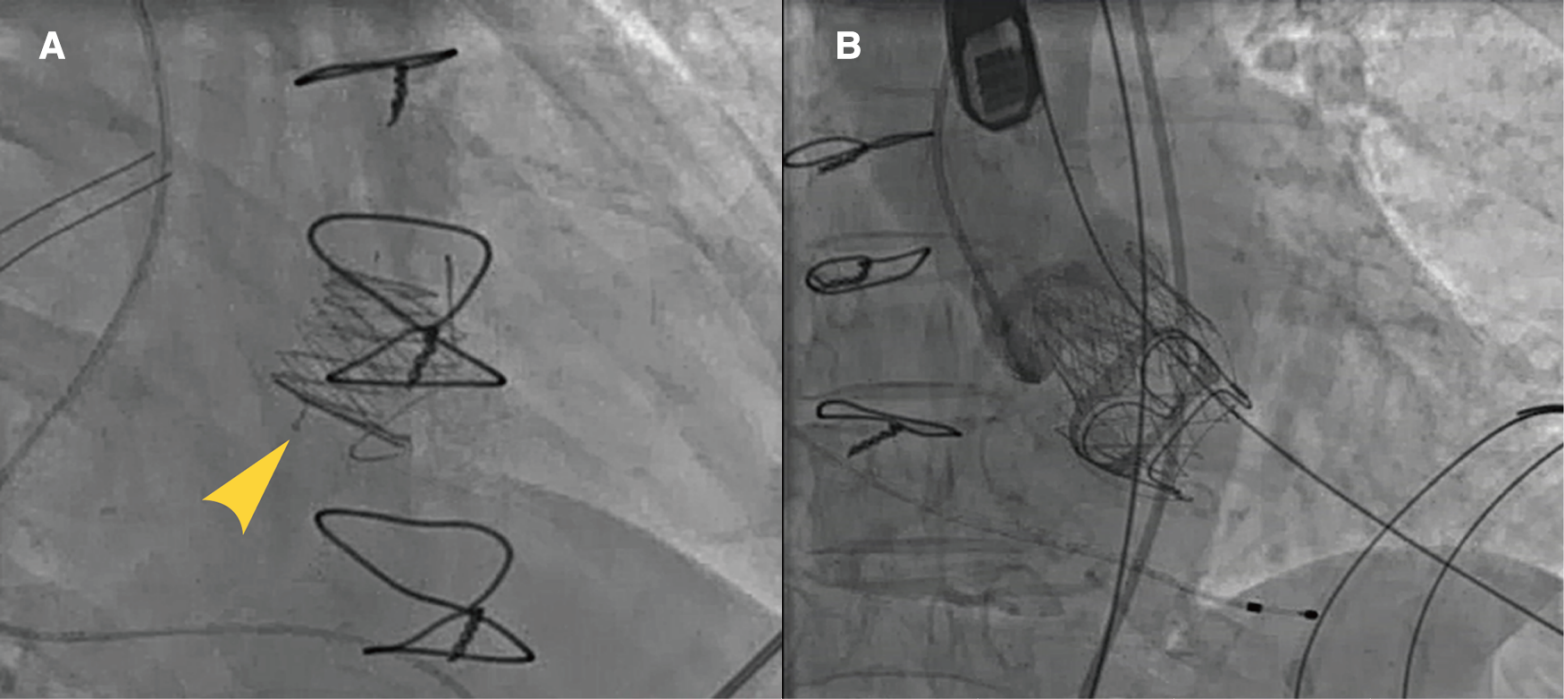
Typical cases of utilization of J-Valve. J-Valve applied to a strut-radiolucent epic valve (St Jude Medical) for ViVTMVR (**A**). Combined aortic valve disease with concomitant TAVR and ViV-TMVR procedures (**B**).

The step-by-step procedure is shown in Figure 2 and Supplementary Video S1. After apical puncture, the failing bioprosthetic mitral valve can usually be crossed easily using J-tip guidewires. A transesophageal echocardiography was used to further confirm the guide wire into the left atrium. The wire was subsequently exchanged for an extra-stiff guide wire with curved tip. Conveyor curvature could be adjusted as needed to provide optimal coaxiality. After entering the left ventricle along the extra-stiff guidewire (Figure 2A), the conveyor first released three U-shaped grippers and subsequently staggered them between three struts of the bioprosthetic valve (Figure 2B). To improve the success and accuracy of this step, a preoperative computed tomography assessment was performed to calculate the C-arm angle at which the tips of the three struts are located at the same level. This is particularly important for the epic valve (St Jude Medical, Inc, St Paul, MN, USA) because its struts are radiolucent (Figure 3A). The valve was slowly released under rapid ventricular pacing (Figure 2C). Subsequently, the conveyor anchor device was controlled to de-load the valve (Figure 2D), the conveyer was withdrawn, and the guidewire retained (Figure 2E). Mitral flow velocity and paravalvular leak were explored using transesophageal echocardiography. Detection of mitral flow velocity and paravalvular leak using transesophageal echocardiography were utilized to determine whether to perform balloon valvuloplasty. The ideal implantation depth was considered to be 80% of the THV stent frame in the left ventricle and 20% in the left atrium (Figure 2F). Coincident native aortic valve disease or prosthetic bioprosthetic valve failure can also be managed concurrently (Figure 3B).

The strategy for postoperative anticoagulation is based on the current guidelines (2, 3) for the management of valvular heart disease and atrial fibrillation (12). Warfarin was administered on the first postoperative day and was continued for 3–6 months. The International Normalized Ratio value was maintained at 2.5. Anticoagulation or antiplatelet therapy was selected depending on the presence or absence of atrial fibrillation and the history of percutaneous coronary intervention with stent implantation or coronary artery bypass surgery.

### Follow-up

All patients were followed up by four researchers (YL, JZ, KW and JS), including telephone interviews and in person visits. Follow-up data included complications reported according to the Valve Academic Research Consortium-2 definition (13), results of transthoracic echocardiography, NYHA functional class for heart failure, and patient-reported health-related quality of life outcome measured by the Kansas City Cardiomyopathy Questionnaire-12 (KCCQ-12) score. The KCCQ-12 score quantitatively assesses the frequency of incident symptoms, physical limitations, social limitations, and quality of life in four areas through 12 questions. The scores can take values between 0 and 100, with higher scores meaning better health status (14).

### Statistical analysis

Continuous variables were expressed as means ± standard deviations (SD) or medians with interquartile ranges, depending on whether they conformed to a normal distribution. Two-sample t-test or Wilcoxon rank sum test was used for comparisons between groups. Categorical variables were expressed as frequencies and percentages. Adverse event rates were based on Kaplan-Meier estimates, and all comparisons were made using the log-rank test. The data were analyzed using SPSS version 26.0 software (SPSS, Chicago, IL, USA). The Kaplan-Meier survival curve and bar chart with error bars were plotted using https://www.bioinformatics.com.cn (last accessed on 31 Oct. 31, 2022), an online platform for data analysis and visualization.

## Results

### Baseline characteristics

Baseline characteristics of the participants are shown in Table 1. Thirty-three consecutive patients underwent a ViV-TMVR procedure in the study cite, with a mean age of 70.1 ± 1.1 years. Thirteen (39.3%) patients were male. The mean time between surgical mitral valve replacement and ViV-TMVR was 10.7 ± 0.6 years. The New York Heart Association functional class was III/IV in 28 patients (84.9%). Surgical bioprosthetic valves included Carpentier-Edwards porcine and pericardial (Edwards Lifesciences, Inc., Irvine, CA, USA), Hancock II and Mosaic (Medtronic, Minneapolis, MN, USA), Epic heart valve (St Jude Medical, Inc, St Paul, MN, USA), and BalMedic bovine pericardial (Balance Medical, Beijing, China). In some patients we were unable to verify the valve type. All patients were subjected to surgical risk assessment, with a mean STS score of 11.8% and a mean (±SD) EuroSCORE II score of 27.4% ± 2.3%. All patients were considered as having high risks associated with conventional surgery.

**Table 1:** Baseline characteristics (*n* = 33).

Characteristic	Value
Male (%)	13 (39.3%)
Age (years)	70.1 ± 1.1
BMI (kg/m^2^)	22.3 ± 0.6
Time to ViV-TMVR from surgical MVR (years)	10.7 ± 0.6
NYHA functional classification Ⅲ/Ⅳ (%)	28 (84.9%)
Chronic obstructive pulmonary disease (%)	4 (12.1%)
Coronary artery disease (%)	9 (27.2%)
Previous coronary artery bypass (CAB) (%)	4 (12.1%)
Prior CVA/TIA (%)	3 (9.0%)
Peripheral vascular disease (%)	5 (15.1%)
Currently receiving dialysis (%)	1 (3.0%)
Diabetes mellitus (%)	6 (18.1%)
Hypertension (%)	14 (42.4%)
Atrial fibrillation (%)	23 (69.6%)
Previous permanent pacemaker (%)	5 (15.1%)
EuroSCORE II	27.4 ± 2.3
STS score	11.8 (7.6, 17.1)

Values are presented as mean ± SD, *n* (%), or median (interquartile range).

ViV TMVR, valve-in-valve transcatheter mitral valve replacement; BMI, body mass index; NYHA, New York Heart Association; CVA/TIA, Cerebrovascular Accident/Transient Ischemic Attack; STS, Society of Thoracic Surgeons.

### Echocardiographic characteristics

Echocardiographic characteristics are presented in Table 2. Preoperative echocardiographic evaluation results showed that seven patients (21%) had stenosis but no regurgitation, 12 patients (36%) had regurgitation but no stenosis, and 14 patients (43%) had both stenosis and regurgitation. The left ventricular ejection fraction and left ventricular size were in the normal range in 28 patients (85%). Mostly combined with moderate to severe tricuspid valve insufficiency. A total of eight patients (24%) had coexisting moderate to severe aortic valve disease. Eight patients (24%) had moderate to severe pulmonary hypertension.

**Table 2:** Preoperative echocardiographic assessment (*n* = 33).

Variable	Value
Mitral valve pathology	
Stenosis (%)	7 (21%)
Regurgitation (%)	12 (36%)
Combined (%)	14 (43%)
LVEF (%)	63 (59, 68)
LVEDd (mm)	45 (43, 50)
LVESd (mm)	30 (28, 32)
LAd (mm)	50 (44, 56)
Peak transvalvular jet velocity (Vmax)(cm/s)	269 ± 49
Tricuspid insufficiency (moderate to severe) (%)	28 (85%)
Combined aortic valve disease	
Aortic insufficiency (moderate to severe) (%)	7 (21%)
Aortic stenosis (moderate to severe) (%)	0 (%)
Combined aortic stenosis with regurgitation (%)	1 (3%)
Combined pulmonary hypertension (moderate to severe) (%)	8 (24%)

Values are presented as mean ± SD, *n* (%), or median (interquartile range).

LVEF, Left Ventricular Ejection Fraction; LVEDd, Left Ventricular End-Diastole diameter; LVESd, Left Ventricular End-Systole diameter; LAd, Left atrial diameter; MVR, Mitral Valve Replacement.

### Intraoperative outcomes

The surgery success rate was 97% according to the definition of the Mitral Valve Academic Research Consortium. One patient's procedure was converted to open-heart surgery due to intraoperative valve embolization to the left ventricle. Transapical access was used for all procedures with the J-Valve™ system. THV sizes ranged from 23 mm (*n* = 11, 33.3%) to 27 mm (*n* = 6, 18.2%), with 25 mm being the most utilized size in a total of 16 patients (48.5%). Pre-dilatation was performed in eight patients (24.2%) and post-dilatation in 10 patients (30.3%) due to a postoperative perivalvular leak. Perivalvular leak or concern about long-term migration due to suboptimal THV release position in two patients (5.1%) were resolved by implanting a second valve. Seven patients (21.2%) were concurrently treated for aortic valvular lesions. The valve in valve transcatheter aortic valve replacement (ViV-TAVR) was concurrently performed in four patients (12.1%) and transcatheter aortic valve replacement (TAVR) in three patients (9.1%).

### Early outcomes

The early clinical outcomes (within 30 days from operation) are shown in Table 3. The median postoperative time to discharge was six days, with no hospital readmissions within 30 days from operation. There were no deaths or other serious complications, except for one patient (2.5%) who experienced stroke. Mild perivalvular leaks occurred in five patients (15.2%). Mitral valve hemodynamics improved postoperatively as demonstrated by the lower transvalvular flow velocity compared to the respective preoperative value (180 ± 9 vs. 269 ± 49 cm/s, *p* < 0.0001).

**Table 3:** Clinical outcomes (*n* = 33).

Outcome	Value
Early outcomes (30 days from the operation)	
All-cause mortality (%)	0 (0.0%)
Cardiovascular death (%)	0 (0.0%)
Duration of hospital stay (days)	6 (5,10)
Readmission within 30 days (%)	0 (0.0%)
Permanent peacemaker required (%)	0 (0.0%)
Complications	
Acute kidney injury (%)	0 (0.0%)
Stroke (%)	1 (2.5%)
Respiratory failure (%)	0 (0.0%)
Left ventricular output tract obstruction (%)	0 (0.0%)
Myocardial infarction (%)	0 (0.0%)
Paravalvular leak	
None (%)	28 (84.8%)
Mild (%)	5 (15.2%)
Moderate to severe (%)	0 (0.0%)
Mitral valve forward flow (cm/s)	179.7 ± 8.9
Peak pressure gradient (mmHg)	11 (8, 15)
Mean pressure gradient (mmHg)	5 (4, 6)
Follow up outcomes (median follow-up was 28 months)	
All-cause mortality (%)	2 (6.1%)
Cardiovascular death (%)	0 (0.0%)
Stroke (%)	2 (6.1%)
Mitral valve reintervention (%)	0 (0.0%)
Myocardial infarction (%)	0 (0.0%)
New dialysis requirement (%)	0 (0.0%)
New pacemaker (%)	0 (0.0%)
NYHA functional classification	
Ⅰ (%)	29 (87.9%)
Ⅱ (%)	2 (6.1%)
Ⅲ/Ⅳ (%)	0 (0.0%)

Values are presented as mean ± SD, *n* (%), or median (interquartile range).

NYHA, New York Heart Association.

### Follow-up outcomes

**Figure 4:**
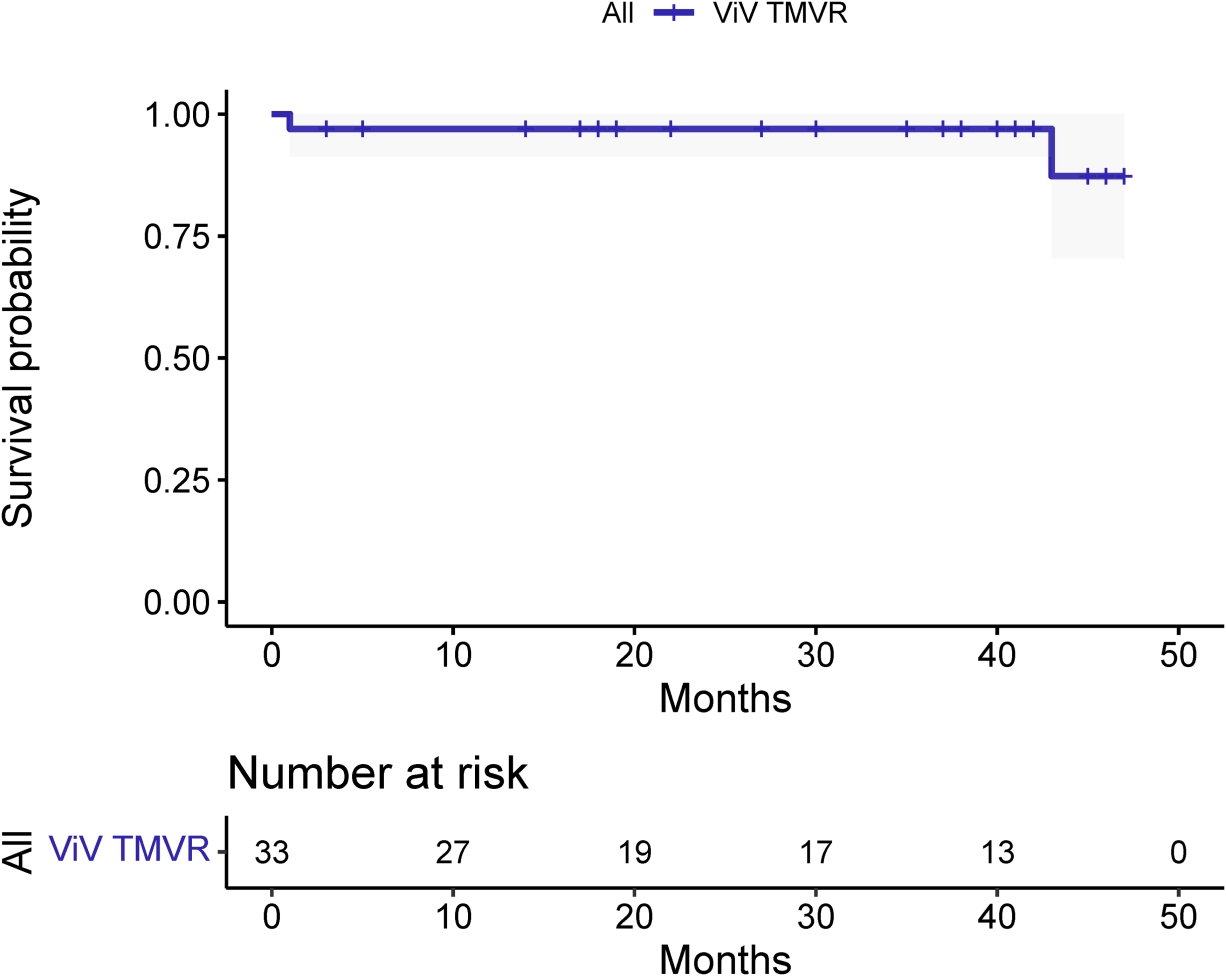
Results of the survival analysis (Kaplan-Meier curve).

**Figure 5:**
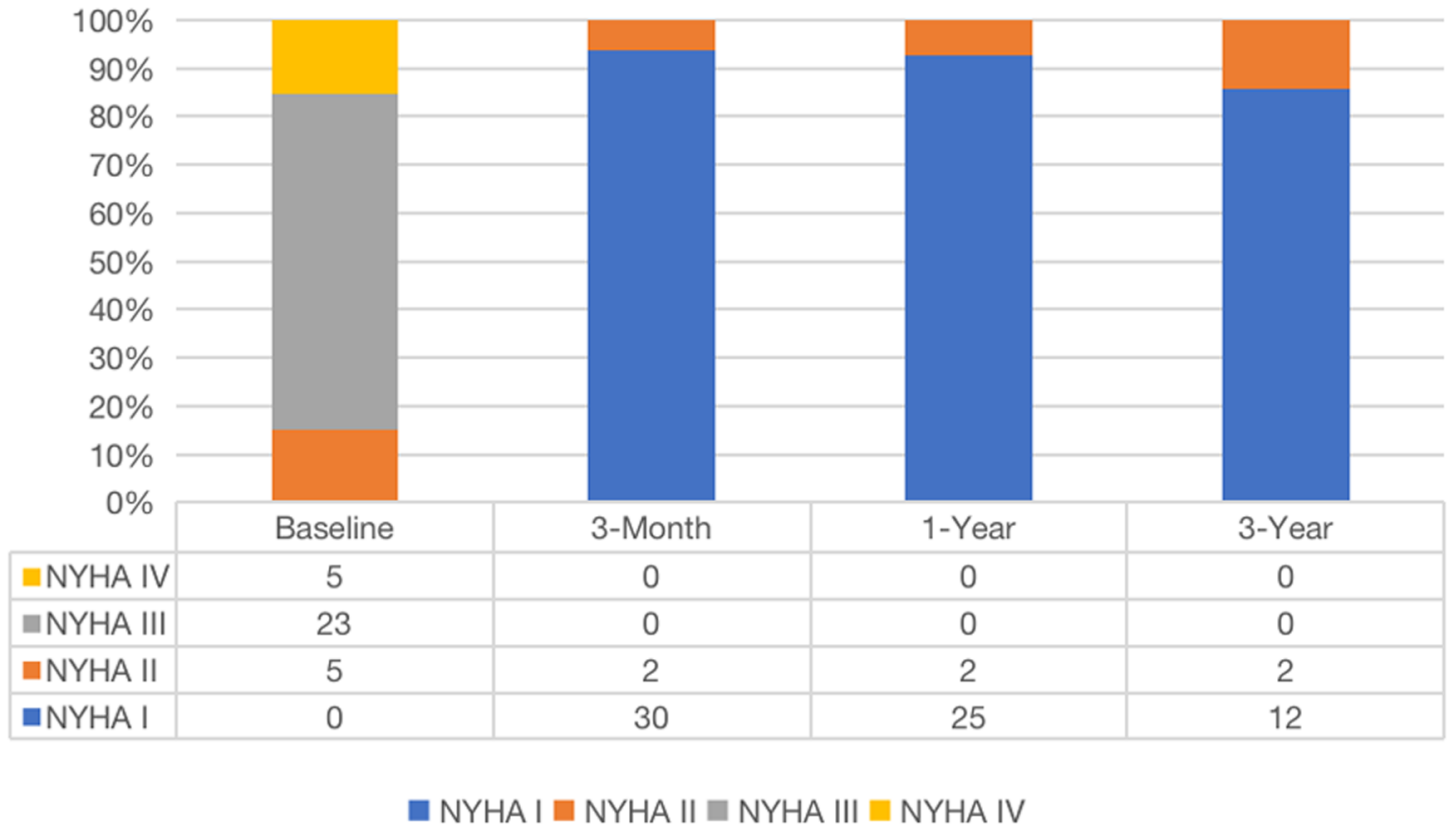
Changes in NYHA classification after surgery.

**Figure 6:**
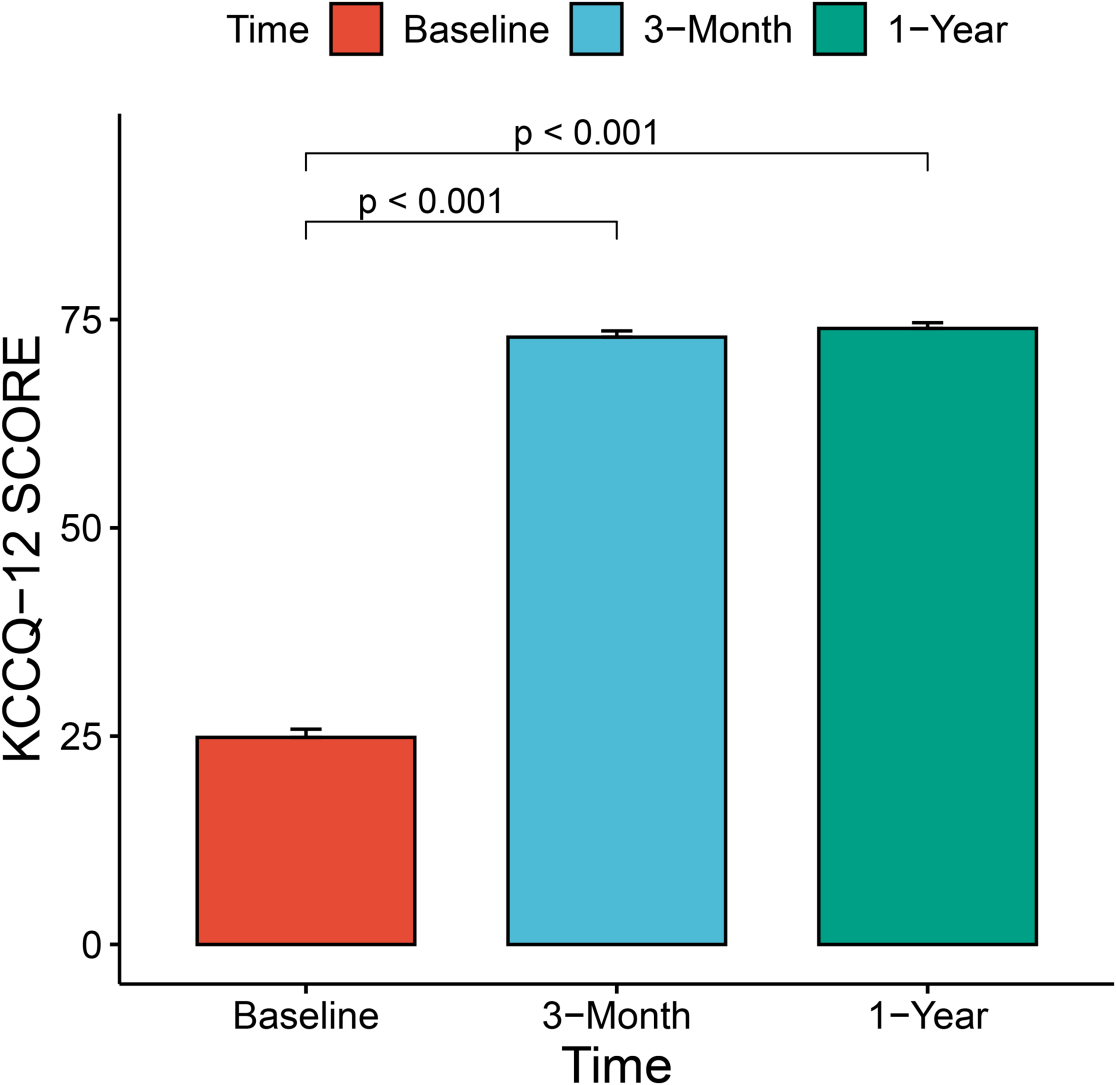
Change in patient health-related quality of life using the Kansas city cardiomyopathy questionnaire-12 score.

The median and maximum follow-up times were 28 and 45 months, respectively. Follow-up outcomes are shown in Table 3. The all-cause mortality was 6.1% (Figure 4): one patient died from pulmonary infection, and another experienced a sudden death for unknown reasons while sleeping at night. No cardiac deaths were recorded. Cerebral infarction occurred in two patients (6.1%): in one patient 10 months after surgery followed by left atrial appendage occlusion performed 7 months later; the other patient failed the ViV-TMVR due to a large left atrium and was converted to direct cardiac surgery with a mechanical prosthetic valve. There were no significant sequelae after thrombolytic therapy. Univariable Cox regression showed that only chronic obstructive pulmonary disease had a significant effect on survival. However, a multivariate Cox regression analysis did not identify any variables that significantly affected survival outcomes. The NYHA classification (Figure 5) and the KCCQ-12 score (Figure 6) were significantly improved when compared to their preoperative values. The mean changes in KCCQ-12 score from baseline three months and one year after the operation were 48.0 (95% confidence interval 46.0, 50.0) and 48.8 (95% confidence interval 47.1, 50.5), respectively (*p* < 0.001).

## Discussion

The standard treatment for bioprosthetic valve failure is redo valve replacement (15, 16). However, studies (4, 6) have shown that ViV-TMVR is associated with lower in-hospital mortality, lower risk of complications, and lower need of resources. Previous studies have reported 30-day mortality rates between 3.2% and 7.5% and one-year mortality rates between 11.3% and 16.9% after ViV-TMVR (17–21). Outcomes from long-term follow-up are less commonly reported (22), with one study reporting a four-year mortality rate of 37.5%, a stroke incidence of less than 3%, and an LVOT obstruction incidence of 0% to 5% (18).

Our study demonstrated good mid-term clinical outcomes and health-related quality of life in patients who received ViV-TMVR, which is clearly better than reported by previous studies. In our study, no patients died immediately after the operation and the all-cause mortality during the follow-up with a median of 28 months was also low (6.1%). No LVOT obstructions were observed either. Mitral valve hemodynamics, NYHA classification and health-related quality of life were also significantly improved in patients after ViV-TMVR. This study used the KCCQ-12 score to reflect patients' health-related quality of life, which is a patient-reported outcome and more accurate than the NYHA classification for detecting changes in health status in patients with heart failure (23).

### The advantages of J-Valve for ViV-TMVR

The J-Valve system consists of a self-expanding transcatheter valve and a transapical interventional device. Several studies (24–27) have confirmed its short- or medium-term safety and efficacy in the treatment of aortic valve disease.

Our center was the first to successfully complete ViV-TMVR using a reverse-loaded J-Valve in January 2019. ViV-TMVR was accomplished in an innovative way by changing the loading direction and release sequence without changing the structure of the J-Valve and conveyors. J-Valve has several advantages when applied to ViV-TMVR. First, its three grippers make leaflet-to-leaflet and commissure-to-commissure positioning simple, without the need to consider commissural misalignment (Figure 1B). The problem of misalignment due to ViV-TMVR has until now been largely ignored in clinical research. Correct orientation is mandatory for surgical bioprosthetic valve replacement (28), which means that commissural posts should not face the LVOT. The risk of LVOT obstruction may be increased if the THV commissure posts point toward the LVOT. There is however no way of preventing misalignment for balloon-expandable THV. Second, J-Valve also has U-shaped notches (Figure 1B) instead of a complete cylindrical metal stent, which minimizes the risk of LVOT obstruction and has particular advantages in patients with small left ventricular volumes (29), meaning that an evaluation of the neo-LVOT is not required (30). Third, because of the fixation of the grippers, the risk of the potentially fatal distant THV migration to the left atrial side (7–10) was reduced. Fourth, J-Valve can be used for both ViV-TAVR and ViV-TMVR, and even for tricuspid bioprosthetic valve failure, which allows valve-in-valve transcatheter tricuspid replacement (ViV-TTVR) using a right atrial approach. Fifth, the self-expanding THV can continuously apply a radial support force on the failed bioprosthetic valve's stents, so that the failed leaflets remain strongly anchored at the frame.

A CT imaging analysis should be considered during preoperative evaluation and planning, especially for evaluating the risk of LVOT obstruction. The two main risk factors for LVOT obstruction are the aortomitral angle and neo-LVOT area (31). The optimal size for the neo-LVOT is unknown, but a minimum of 200–250 mm^2^ has been suggested (32). In our study, LVOT obstruction has to our knowledge been never detected with postoperative TEE. Therefore, preoperative assessment of the risk of LVOT obstruction appeared to be unnecessary for J-Valve when ViV-TMVR was performed. The possibility to avoid CT imaging simplifies the pre-operative assessment procedure, demonstrating a further advantage of the J-Valve structure.

In addition, in our study, contrast agent were not needed to be used during the whole operation. This benefits patients with allergic asthma, abnormal thyroid function, and chronic renal insufficiency, and can reduce the risk of perioperative complications for such patients.

### Surgical approach

The approach to ViV-TMVR can be divided into surgical access and complete percutaneous access. The corresponding approaches are transapical and transseptal, respectively. Currently, transapical approach is by far the most common way for transcatheter valve implantation in the mitral position. Updated data from the Valve-in-Valve-International-Data (VIVID) registry shows that the transapical approach is utilized in 81% of valve in valve cases and 68% in valve in ring cases (33). The proximity of the apex to the mitral valve allows for better control of the position of the delivery device with better coaxiality, does not require many guidewires and sheaths, and is suitable for surgeons with limited experience in performing this intervention. Attention should be drawn to the fact that transcatheter apical-related complications include not only the impairment of left ventricular apical function (34, 35), but also pleural effusion, bleeding, atrial fibrillation, and prolonged intubation time (36–39). Transseptal mitral valve implantations are becoming more common worldwide, and have the main advantage of being less invasive and not requiring open surgery or left ventricular trauma; unfortunately, the device has poor coaxiality with the mitral orifice plane. In addition, transseptal access requires puncture of the atrial septum and balloon atrial septostomy, which remains technically challenging and may present complications such as iatrogenic atrial septal defect (iASD), cardiac perforation and tamponade (40). Consequently, the transseptal approach is mainly suitable for surgeons with rich intervention experience.

In our study, only the transapical approach was used. The J-Valve system provides the most direct, shortest, and most coaxial access to the mitral valve. The transapical approach also enables treatment of aortic valve diseases or mitral perivalvular leak occlusion while performing ViV-TMVR. Seven patients in our study were treated for aortic valvular lesions and one patient for mitral perivalvular leak occlusion simultaneously while performing ViV-TMVR. From our experience, the transapical approach for simultaneous ViV-TMVR and ViV-TAVR appears to be operationally more convenient, allowing sequential release of both THVs from the same puncture site. In addition, none of the patients in this study experienced postoperative apical bleeding or complications such as guidewire-related cardiac injury, demonstrating the good safety of the transapical approach.

### Limitations and future directions

The present investigation was a real-world, retrospective clinical study, which consequently comes with the limitations of an observational study. First, the study was conducted in a single center, so the selection of the patients may have been biased and the results are not necessarily generalizable for broader populations; however, the study population was enrolled consecutively to minimize selection bias. Second, considering that there are no long-term results of J-Valve for ViV-TMVR, we only performed this surgery on patients who are elderly, high-risk or surgically contraindicated, which resulted in a small sample size. Longer-term follow-up data are therefore needed. Third, in the absence of an echocardiographic core laboratory, echocardiographers were able to only determine the presence or absence of left ventricular outflow tract obstruction without recording the outflow tract flow velocities, making it impossible to track the values of the related variables or to compare differences in preoperative and postoperative outflow tract flow velocities. Fourth, some outcome measures were patient-reported, which may also cause bias.

Future multicenter clinical trials are needed to validate the safety and efficacy of the surgical approach addressed in our study. Studies that have sufficiently large sample sizes and long follow-up duration, and that include a control group, are needed also to identify the factors independently associated with survival.

## Conclusion

This study demonstrates that J-Valve system is a safe and effective option for ViV-TMVR: it has a high success rate and low mortality, and resulted in very few complications. Mitral valve hemodynamics, NYHA classification and health-related quality of life also significantly improved in patients after ViV-TMVR with J-Valve. The innovative use of the J-Valve for ViV-TMVR is a promising alternative surgical option for the elderly, high-risk patients with bioprosthetic mitral valve failure. Future multicenter clinical trials with long-term follow-up are however needed to strengthen the evidence on the safety and efficacy of this surgical approach.

## Data Availability

The raw data supporting the conclusions of this article will be made available by the authors, without undue reservation.

## Supplementary material

SUPPLEMENTARY VIDEO S1

Animated demonstration of applying J-Valve to ViV-TMVR surgical procedure.
