# Supplementary figures and images for: Innovative use of a self-expanding valve for valve-in-valve transcatheter mitral valve replacement: experience from a four-year single-center study
Source: Front Cardiovasc Med. 2023 Jun 12;10:1137663. doi: 10.3389/fcvm.2023.1137663 (PMC10292798; doi:10.3389/fcvm.2023.1137663)

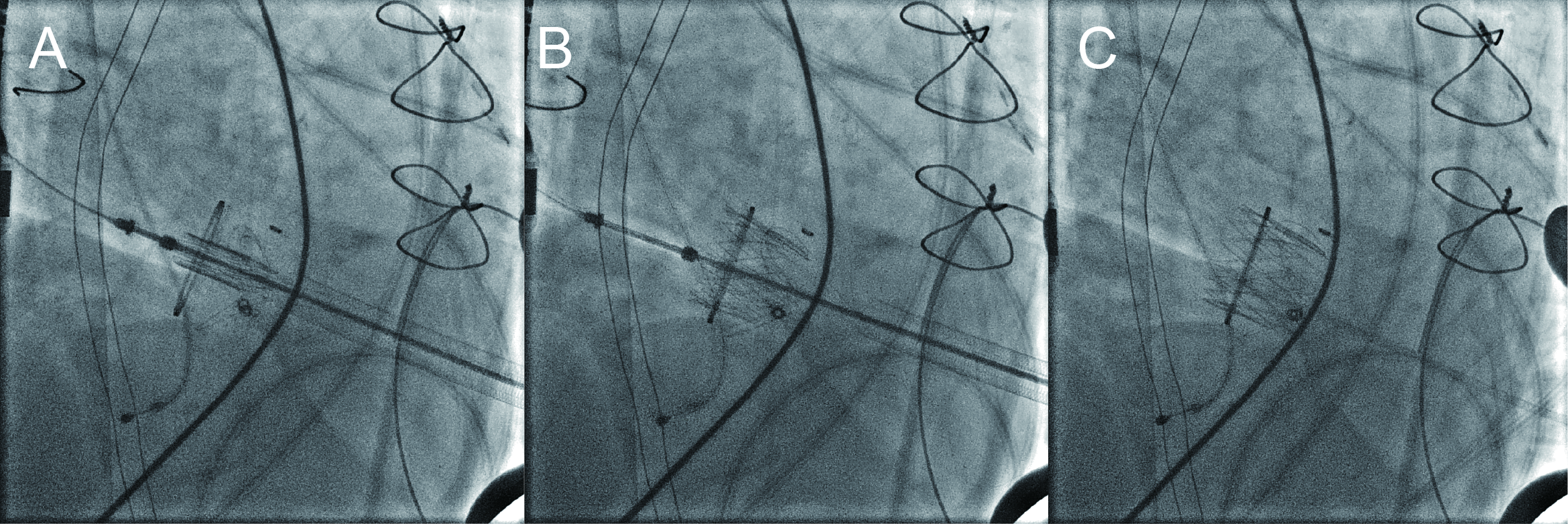

Supplement: Supplementary file 1 [file Image1.tif]
